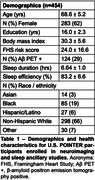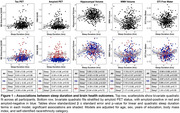# Associations between imaging biomarkers and sleep quality in the U.S. POINTER study baseline cohort

**DOI:** 10.1002/alz.090450

**Published:** 2025-01-09

**Authors:** Joseph R. Winer, Tyler J. Ward, Margaret Scales, Jacinda Taggett, Marjorie Howard, Katie L Stone, Samuel N. Lockhart, Laura D Baker, Kathleen M. Hayden, Susan M. Landau, Theresa M. Harrison, The U.S. POINTER Study Group

**Affiliations:** ^1^ Stanford University School of Medicine, Stanford, CA USA; ^2^ University of California, Berkeley, Berkeley, CA USA; ^3^ Wake Forest University School of Medicine, Winston‐Salem, NC USA; ^4^ University of California, San Francisco, San Francisco, CA USA; ^5^ California Pacific Medical Center Research Institute, San Francisco, CA USA; ^6^ Wake Forest University, Winston‐Salem, NC USA; ^7^ Wake Forest University School of Medicine, Winston Salem, NC USA

## Abstract

**Background:**

Worse sleep is associated with a greater risk of Alzheimer’s disease (AD). However, few studies have linked objective sleep with neuroimaging outcomes, limiting our understanding of how poor sleep impacts the brain. The U.S. POINTER trial, which has enrolled a heterogeneous cohort of older adults at elevated risk for cognitive decline, is a unique sample differing from prior investigations of sleep, aging, and brain health. Here we investigate how actigraphy‐assessed sleep duration and efficiency are cross‐sectionally associated with baseline neuroimaging measures of brain structure and AD pathology.

**Method:**

Participants underwent neuroimaging (MRI, 18F‐florbetaben amyloid PET, and 18F‐MK6240 tau PET), cognitive testing, and wristwatch actigraphy, which assessed sleep duration and sleep efficiency averaged across six nights. Imaging analyses focused on bilateral entorhinal tau PET, cortical amyloid PET, hippocampal volume, white matter hyperintensity (WMH) volume, and diffusion free water. Based on prior literature suggesting short and long sleep duration are disadvantageous, quadratic models were fit for sleep duration predicting neuroimaging and cognitive measures, with age, sex, years of education, body mass index, and self‐identified racial ethnic category as covariates. Linear models were fit for sleep efficiency with the same covariates.

**Result:**

Across 454 participants (Table 1), sleep measures were not related to cognition. Hippocampal volume, WMH volume, and free water showed U‐shaped relationships with sleep duration such that average sleep durations shorter and longer than approximately 6‐7 hours were associated with worse brain health outcomes (Figure 1). There were no associations between tau or amyloid PET and sleep duration. When stratified by amyloid status, associations between sleep duration and brain structure were significant in only the amyloid negative group. Unexpectedly, there were no associations between neuroimaging measures and sleep efficiency except for a significant positive relationship with entorhinal tau PET uptake.

**Conclusion:**

In the largest and most heterogeneous sample to date of older adults with actigraphy and neuroimaging measures, sleep duration was associated with multiple measures of brain structure but not AD pathology. These relationships may reflect heterogeneity and selection characteristics of the at‐risk POINTER cohort, which differ from previous studies of sleep and AD biomarkers.